# Vaccination and Tick-borne Encephalitis, Central Europe

**DOI:** 10.3201/eid1901.120458

**Published:** 2013-01

**Authors:** Franz X. Heinz, Karin Stiasny, Heidemarie Holzmann, Marta Grgic-Vitek, Bohumir Kriz, Astrid Essl, Michael Kundi

**Affiliations:** Author affiliations: Medical University of Vienna, Vienna, Austria (F.X. Heinz, K. Stiasny, H. Holzmann, M. Kundi);; National Institute of Public Health, Ljubljana, Slovenia (M. Grgic-Vitek);; National Institute of Public Health, Prague, Czech Republic (B. Kriz); and GfK Austria Healthcare, Vienna (A. Essl)

**Keywords:** tick-borne encephalitis, TBE virus, TBE epidemiology, TBE vaccination, field effectiveness of TBE vaccination, parasites, vector-borne infections, viruses

## Abstract

Tick-borne encephalitis (TBE) is a substantial public health problem in many parts of Europe and Asia. To assess the effect of increasing TBE vaccination coverage in Austria, we compared incidence rates over 40 years for highly TBE-endemic countries of central Europe (Czech Republic, Slovenia, and Austria). For all 3 countries we found extensive annual and longer range fluctuations and shifts in distribution of patient ages, suggesting major variations in the complex interplay of factors influencing risk for exposure to TBE virus. The most distinctive effect was found for Austria, where mass vaccination decreased incidence to ≈16% of that of the prevaccination era. Incidence rates remained high for the nonvaccinated population. The vaccine was effective for persons in all age groups. During 2000–2011 in Austria, ≈4,000 cases of TBE were prevented by vaccination.

## Abstract 2

Tick-borne encephalitis is a disease of the brain caused by a virus found in many parts of Europe as well as central and eastern Asia. As the name indicates, the virus is spread by tick bites. The number of people infected each year varies according to complex interactions involving the ticks’environment, the weather, and human socioeconomic and vaccination status. To determine how well vaccine protects against the disease, researchers compared the number of cases in 3 neighboring countries in which vaccination coverage differs but many other factors remain the same: Austria (where more than three quarters of the population are vaccinated) and Slovenia and the Czech Republic (where less than one quarter of the population are vaccinated). They found far fewer cases in Austria, indicating that vaccination is an excellent way to prevent this disease.

Tick-borne encephalitis (TBE) is the most common arthropod-transmitted viral infection of humans in Europe and central and eastern Asia (*1*); each year, >10,000 TBE patients are hospitalized. The role of TBE as a travel-associated disease is probably underestimated (*2*,*3*). TBE virus is a member of the family *Flaviviridae*, genus *Flavivirus*, and a close relative of the mosquito-transmitted viruses that cause yellow fever, dengue fever, Japanese encephalitis, and West Nile fever (*4*). Three antigenically closely related subtypes are carried primarily by *Ixodes ricinus* (European subtype) and *I. persulcatus* ticks (Siberian and Far-Eastern subtypes) (*5*). In TBE-endemic areas, the virus circulates between ticks and vertebrate hosts (primarily rodents) (*5*,*6*); humans are dead-end hosts only and do not play any role in the maintenance of TBE virus in nature. In most instances, transmission to humans occurs by the bites of infected ticks; however, in some TBE-endemic areas, alimentary infections—obtained through consumption of raw milk or milk products from infected goats, sheep, or cattle—are common (*6*,*7*). Because virus circulation depends on an intricate balance of virus and host factors that are controlled by environmental conditions, TBE-endemic areas do not follow all areas of tick infestation but are restricted to certain regions that are conducive to maintenance of natural virus cycles (*8*–*11*). In Europe, the most strongly affected countries are southern Germany, Switzerland, Austria, the Czech Republic, Slovakia, Hungary, Slovenia, the Baltic countries, Poland, parts of Scandinavia, and European Russia.

Similar to other flavivirus infections, only a subset of TBE virus infections leads to neurologic diseases such as meningitis, encephalitis, encephalomyelitis, and radiculitis (*12*). On average, the severity of disease increases with patient age (*13*), and case-fatality rates of <1%, 1%–3%, and <35% have been reported in Europe, Siberia, and the Far East, respectively (*12*). Effective inactivated whole virus vaccines are produced in Europe (European subtype strain) and Russia (Far-Eastern subtype strain) (*1*), but their usage differs widely among TBE-endemic countries (*14*,*15*). Experiments with postvaccination serum and direct mouse challenge experiments have shown that vaccines manufactured with 1 subtype will also protect against strains of the other TBE virus subtypes (*16*,*17*), consistent with their antigenic similarity. A strong upsurge of TBE in Europe in recent years (*6*) has been associated with climatic, ecologic, and human behavioral changes that might increase the risk for virus exposure (*8*,*18*–*20*).

To determine the effectiveness of vaccination, we examined incidence of TBE in Austria over 40 years, including 10 years without vaccination followed by 30 years with increasing vaccination coverage. We compared these data with those for the Czech Republic and Slovenia, 2 neighboring central European countries with high TBE incidence rates but comparatively low vaccination rates. We demonstrate that the strong decline of TBE observed only in Austria resulted from protection by vaccination and that the incidence rate for the nonvaccinated population remained as high as it was during the prevaccination era. The data from all 3 countries reveal a strong degree of annual and longer range variations, which are coincident in some but not all instances.

## Materials and Methods

### TBE Vaccines and Vaccination Schedules

The TBE vaccines available on the European market are produced by 2 manufacturers: FSME-IMMUN by Baxter AG, Vienna, Austria, and Encepur by Novartis Vaccines, Marburg, Germany. Both vaccines contain purified TBE virus grown in chick embryo cells, inactivated by formalin, and have aluminum hydroxide added as adjuvant (*21*). In Austria, the recommended basic vaccination schedule consists of 2 vaccinations ≈4 weeks apart followed by a third vaccination after 5–12 months and a fourth vaccination after >3 years. For persons <60 years of age, additional booster immunizations are recommended every 5 years; this interval is reduced to 3 years for persons >60 years of age. Both manufacturers also provide vaccines for children; these vaccines contain half of the antigen dose contained in the vaccines for adults (*22*,*23*).

### Collection of Vaccination Coverage Data for Austria

For Austria, TBE vaccination status data were collected annually by postal surveys conducted by GfK Austria Health Care (Vienna, Austria); 4,000 households (8,500–10,000 household members, representative of different age groups) were surveyed. Data were acquired from written vaccination history for 87% of participants and memory for 13%.

### Documentation of TBE Cases

For several decades, Austria, the Czech Republic, and Slovenia have had well-established systems for documenting TBE cases (*20*,*24*,*25*), and, according to the National Reference Laboratories and/or National Public Health Institutes, the principles of the notification system were not changed over the period analyzed in this study. For all 3 countries, incidence rates refer to cases confirmed by laboratory diagnosis. This confirmation is based on TBE virus IgM and IgG ELISA results, which replaced the hemagglutination-inhibition and/or complement fixation assays used until the early 1980s in Austria and the early 1990s in the Czech Republic and Slovenia. In Austria, data are collected by the Department of Virology of the Medical University of Vienna, which serves as a national reference laboratory for TBE virus and other flaviviruses. The documentation includes the history of vaccination; for the purposes of this study, participants were subdivided into groups: those who followed the regular schedule of vaccination and those who had received an undefined number of vaccinations outside the recommended schedule. For a small (5%) proportion of TBE patients, no precise information about vaccination status could be obtained.

### Calculation of Field Effectiveness of Vaccination in Austria

Calculation of field effectiveness of vaccination was based on TBE incidence and vaccination coverage for different age groups as described (*24*). For significance testing, a Monte Carlo procedure was chosen; 10,000 samples were collected under the zero hypothesis of no difference in vaccination effectiveness. p values were determined as the percentiles of the obtained distributions.

Because vaccination status remained undefined for 5% of the 883 TBE patients in Austria during 2000–2011, we analyzed best-case and worst-case scenarios for vaccine effectiveness. For the best-case scenarios of regularly and irregularly vaccinated patients, 45 patients with unknown vaccination history were omitted; for the worst-case scenarios, these patients were assumed to have been regularly or irregularly vaccinated, respectively.

### Calculation of Incidence Rates 

Population data for the calculation of incidence rates were obtained from Statistics Austria (www.statistik.at/web_en/), the Czech Statistical Office (www.czso.cz/eng/redakce.nsf/i/home), and the Statistical Office of the Republic of Slovenia (www.stat.si/eng/index.asp). For the analysis of time trends, we used a piecewise log-linear model, the joinpoint analysis, to identify possible trend changes during 1972–2011 (Joinpoint Regression Program, version 3.5.2; Statistical Research and Applications Branch, National Cancer Institute, Silver Spring, MD, USA). The model was specified to include a maximum of 5 joinpoints, constrained to be at least 2 years apart and at least 2 years from the start and end of the entire period. The best fitting model was searched under the assumption of a Poisson variation of the number of patients and the population size in midyears as the offset variable. These analyses were conducted separately for the Czech Republic, Slovenia, and Austria. For Austria, these analyses were conducted for the overall incidence and for the incidence among nonvaccinated persons. For the years before 2000, the incidence among nonvaccinated persons was estimated by assuming a vaccine effectiveness of 97%; from 2000 on, the actual number of cases that occurred among nonvaccinated persons was used for the calculation. 

## Results

### Incidence of TBE in Austria, the Czech Republic, and Slovenia

**Figure 1:**
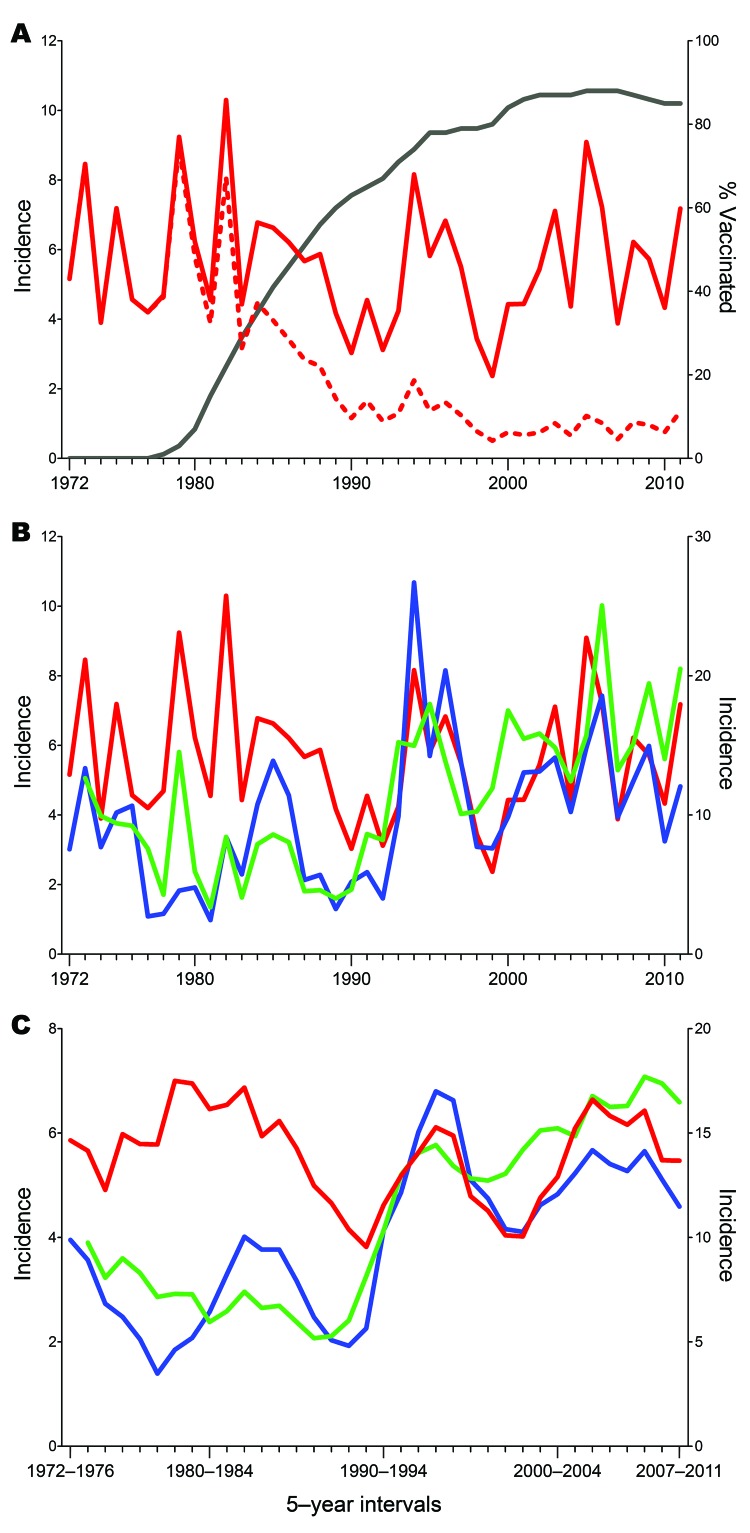
Tick-borne encephalitis (TBE) incidence rates, 1972–2011, central Europe. A) Total population (red dashed line) and nonvaccinated population (red solid line) in Austria. The black line represents the increasing coverage of vaccination, which started in 1978. B) Comparative representation of TBE incidences in Austria (red line), Czech Republic (green line), and Slovenia (blue line). The incidence scale for Slovenia (right y-axis) differs from that of Austria and the Czech Republic (left y-axis). C) Sliding-window representation of TBE incidence in Austria (red line), Czech Republic (green line), and Slovenia (blue line) in means of 5-year intervals. The incidence scale for Slovenia (right y-axis) differs from that of Austria and the Czech Republic (left y-axis).

Epidemiologic data on TBE in Austria have been available since 1972; since then, 8,493 cases have been reported through 2011. The corresponding annual incidence rates are displayed in Figure 1, panel A. In the first 10 years, the average incidence was 5.7 cases per 100,000 population (range 3.9­­–9.0), after which (past 10 years) incidence declined dramatically to an average incidence of 0.9 cases per 100,000 population (range 0.6–1.3). This decline was coincident with the increasing rate of TBE vaccination that started in 1972, reached 88% in 2005 (i.e., 88% of the total population had received >1 doses of TBE vaccine), and slightly leveled off to 85% in 2011. Calculation of the incidence rates for the nonvaccinated population (Figure 1, panel A) revealed an average of 6.0 cases per 100,000 population in the past 10 years (comparable to the prevaccination era) and similar extents of annual variation (range 3.9–9.1), demonstrating that the overall decline resulted not from a lower risk for infection but indeed from protection by vaccination.

In the Czech Republic, 18,196 cases were reported during 1973–2011; in Slovenia, 8,129 cases were reported during 1970–2011. Figure 1, panel B, shows a comparison of the annual TBE incidence rates among persons in these 2 countries with those of the nonvaccinated population in Austria. For each of the 3 countries, the patterns vary markedly, and the degree of coincidence of these variations does not seem to follow a distinct annual pattern. For example, the 2 peaks in 1994 and 1996 coincide for Austria and Slovenia but not for the Czech Republic. Conversely, coincident peaks for Slovenia and the Czech Republic were observed in 2006 and 2009; for Austria, they were shifted to the preceding year. However, for all 3 countries, coincident declines (2004, 2007, 2010) and increases (2011) also occurred.

**Figure 2:**
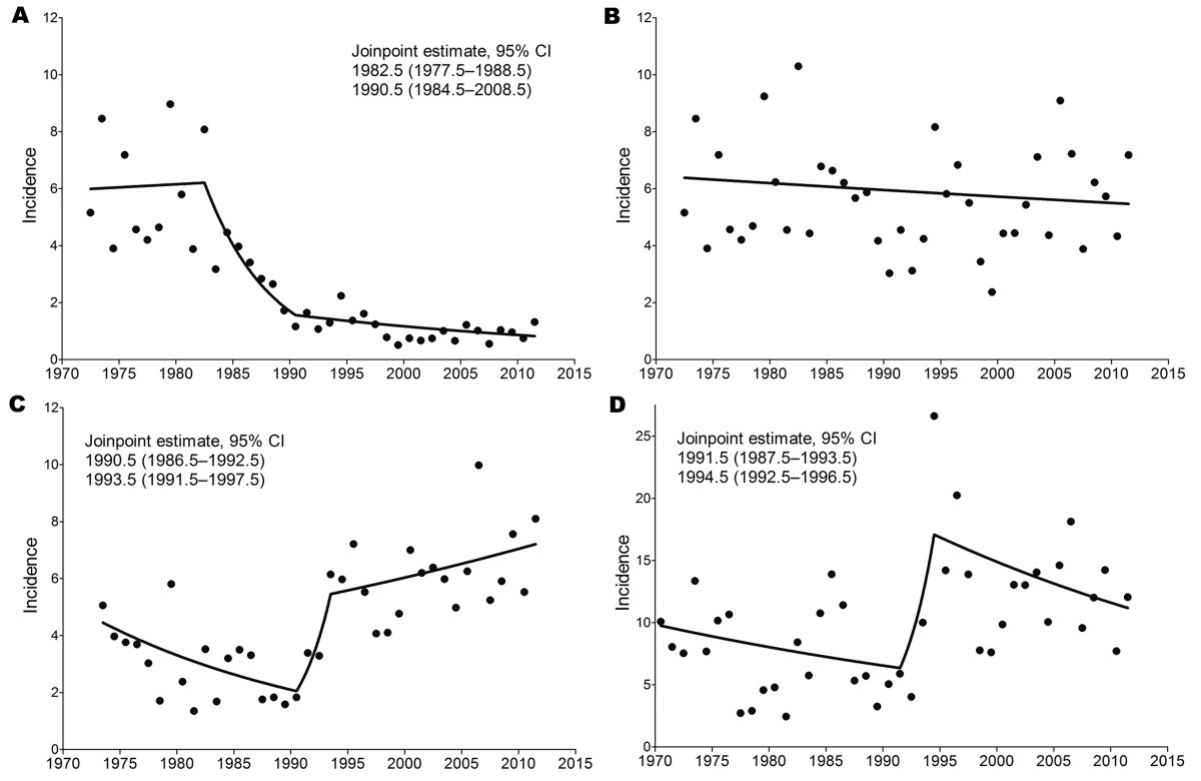
Results of joinpoint analysis of annual incidence rates (no. cases/100,000 population) of tick-borne encephalitis (TBE) in A) Austria (total population), B) Austria (nonvaccinated population), C) Czech Republic, and D) Slovenia. The lines in each panel represent the piecewise log-linear relationship between year and incidence. Estimated joinpoints and their 95% CIs are shown.

For better visualization of longer range temporal changes in incidence rates, we used a sliding-window representation of the means of 5-year intervals (Figure 1, panel C). As described (*20*,*25*) and confirmed by joinpoint analysis, strongly increased incidence was noted for the Czech Republic (starting in 1992; Figure 2, panel C) and for Slovenia (starting in 1993; Figure 2, panel D), whereas no such increase was detected for Austria, at least as determined from the incidence rates for the nonvaccinated population (Figure 2, panel B). The sliding-window representation (Figure 1, panel C), however, suggests longer ranging factors that lead to parallel changes of incidence rates for all 3 countries, as exemplified by the relative declines around 1986–1992, 1995–2003, and 2003–2004 and the relative increases around 1993–1999, 2006, and 2009. A clear discrepancy, however, was observed between incidence rates for Austria and Slovenia and incidence rates for the Czech Republic during 1996–2003.

### Age of TBE Patients 

**Figure 3:**
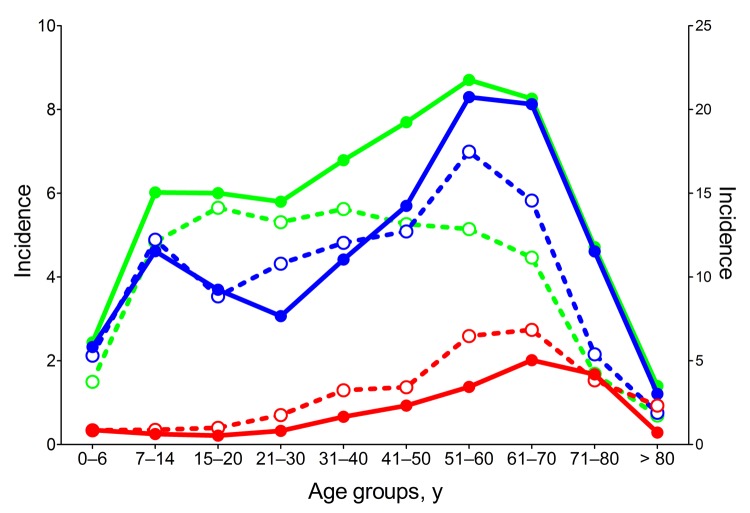
Age distribution of tick-borne encephalitis (TBE) patients during 1990–1999 (dotted lines; open symbols) and 2000–2010 (solid lines; closed symbols) in Austria (red), Czech Republic (green), and Slovenia (blue). The incidence scale for Slovenia (right y-axis) differs from that of Austria and the Czech Republic (left y-axis).

The severity of TBE tends to increase as age increases, thereby resulting in a higher proportion of encephalitis cases relative to meningitis cases in older than in younger persons (*13*). Independent of undefined age-specific physiologic characteristics that control disease severity, the risk for virus exposure might differ among age groups, depending on behavioral factors. Analysis of the age distribution of TBE patients in the 3 countries during 1990–1999 and 2000–2010 revealed significant differences (Figure 3). In all 3 countries, incidence rates were highest among elderly persons. During the past decade, incidence rates increased sharply among those 50–80 years of age in the Czech Republic and Slovenia but not in Austria. The substantial TBE incidence rates among children and adolescents in Slovenia and the Czech Republic have virtually disappeared in Austria, although high rates were typical during the prevaccination era (*26*).

### Field Effectiveness of Vaccination

Using data on vaccination coverage in Austria and TBE incidence rates for nonvaccinated and vaccinated populations, we calculated the field effectiveness of vaccination for 2000–2011. Patients were stratified into 3 groups: those with a documented history of no vaccination, those vaccinated according to regular schedule (i.e., according to recommendations in Austria), and those vaccinated on an irregular schedule. For some (5%) TBE patients, we were unable to obtain accurate information on their vaccination history and therefore made a worst-case calculation by assuming that all of these patients had been vaccinated according to the recommended schedule. The Table shows that the overall field effectiveness for regularly vaccinated persons is ≈99% under best-case assumptions and 96% under worst-case assumptions. The rate of vaccination failure—usually characterized by a delayed IgM and an anamnestic IgG response (*27*)—was not enhanced among elderly persons, indicating that the vaccine-induced immune response of elderly persons was sufficient to prevent disease in most instances, despite the fact that titers were lower among elderly than among younger vaccinees (*28*). Irregularly vaccinated persons have a lower degree of protection: 92.5% protection in the best-case and 91.3% in the worst-case scenarios. Vaccine effectiveness seems to be somewhat lower among younger children than among persons >15 years of age, but statistical significance is reached only under best-case assumptions after regular vaccination. Because the number of cases in this group was low (only 10 in the entire 12 years: 7 among those 0–6 years of age and 3 among those 7–14 years of age), further stratification yielded extremely wide and overlapping 95% CIs that do not enable meaningful conclusions to be drawn.

**Table:** Field effectiveness of TBE vaccination, Austria, 2000–2011*

Scenario, by age group, y	Unvaccinated persons		Regularly vaccinated persons			Irregularly vaccinated persons	
	Incidence†		Incidence†	FE, % (95% CI)		Incidence†	FE, % (95% CI)
Best-case‡							
0–14	1.62		0.10	94.0 (88.0–97.0)		0.18	88.6 (63.3–96.5)
15–50	5.41		0.02	99.7 (99.3–99.9)		0.26	95.3 (92.9–96.9)
51–60	7.60		0.13	98.2 (96.7–99.1)		0.31	96.0 (91.4–98.1)
>61	7.52		0.14	98.2 (97.0–98.9)		0.63	91.7 (87.9–94.3)
Total	5.01		0.07	98.7 (98.2–99.0)		0.37	92.5 (90.3–94.3)
Worst-case§							
0–14	1.62		0.13	92.2 (87.4–97.0)		0.19	88.0 (62.3–96.2)
15–50	5.41		0.11	98.1 (97.4–98.8)		0.30	94.5 (91.9–96.2)
51–60	7.60		0.25	96.8 (95.3–98.4)		0.39	94.9 (89.9–97.4)
>61	7.52		0.44	94.4 (92.8–96.1)		0.71	90.5 (86.4–93.3)
Total	5.01		0.20	96.3 (95.5–97.0)		0.44	91.3 (88.9–93.2)

*TBE, tick-borne encephalitis; FE, field effectiveness. †Cases/100,000 population. ‡Persons with TBE but unknown vaccination status were excluded. §Persons with TBE but unknown vaccination status were considered regularly vaccinated**.**

Taking TBE incidence rates among nonvaccinated persons as a basis, 5,242 cases would have been expected in the absence of vaccination during 2000–2011. However, because the observed number was only 883, it can be concluded that >4,000 cases have been prevented by vaccination in Austria.

## Discussion

A characteristic feature of the epidemiology of TBE is that incidence of TBE among humans in disease-endemic regions can vary dramatically from year to year; in the central European countries analyzed in this study, 2-fold to 3-fold annual variations were found. The reasons for these extensive fluctuations are complex and reflect an intricate interplay of factors that control the natural cycle and transmission dynamics of TBE virus. Climatic and ecologic factors influence the population dynamics of ticks and their vertebrate hosts, such as rodents and larger mammals (*5*,*6*), thereby affecting the abundance of ticks, especially infected ticks, in different years and different regions. A crucial prerequisite for establishing and maintaining TBE virus in its natural cycle seems to be a microclimate that favors the temporal synchrony of larvae and nymph development in spring and that thus enables transstadial transmission of the virus through cofeeding of infected nymphs and uninfected larvae on the same rodent host (*29*,*30*). These peculiar requirements also help explain why TBE virus is not endemic to many parts of Europe where Lyme borreliosis, which is transmitted by the same ticks, occurs (*31*). Even in regions where both agents cocirculate, transmission of Lyme borreliosis seems to follow tick habitats wherever they occur, whereas TBE is confined to only a subset of these locations (*9*). In addition to ecologic factors, specific weather conditions and socioeconomic changes can affect persons’ outdoor activities, leading to enhanced or diminished risk for exposure to TBE virus–infected ticks (*8*,*18*). It is, however, unlikely that the increases and decreases in incidence rates observed from year to year in central Europe result from socioeconomic changes because such changes would not be expected to fluctuate so strongly on an annual basis. Also, similar extents of annual incidence fluctuations are observed in most other countries where TBE virus is endemic (*32*), suggesting that these are driven primarily by biological and climatic factors.

In addition to the short-term fluctuations, our data analysis also revealed longer range undulations of incidence rates in intervals of >5 years, which seemed to be parallel in the 3 countries to a substantial extent (Figure 1, panel C). Except for the strong overall upsurge of TBE cases in the Czech Republic and Slovenia (but not in Austria) starting around 1992, the long-range incidence curves for 1990–2011 are remarkably similar for all 3 countries, suggesting substantial covariation of the underlying risk-for-exposure conditions. The upsurge of TBE in several European countries around 1993 might result from several factors (*8*,*18*); however, all factors that influence the dynamics of tick development and human outdoor activities would be expected to have similar effects on the incidence of Lyme borreliosis (*5*,*9*,*31*). Recent data from Latvia, however, clearly demonstrate an uncoupling of the incidence rates for the 2 diseases (*33*). The decrease of TBE cases after 1998 was not matched by the increasing incidence of Lyme borreliosis or by the unchanged high activity of ticks (*33*). These data, therefore, support the assumption that, in addition to tick vector dynamics and risk for exposure to tick bites in general, the specific factors controlling circulation of TBE virus in its natural hosts (*8*) have a major effect on the long-range characteristics of TBE endemicity. A significant increase was not detected in the overall incidence of TBE in Austria in the 40-year observation period (Figure 1, panel C), in contrast to the upsurges seen in the Czech Republic and Slovenia at the beginning of the 1990s. However, incidence rates among the nonvaccinated population in Austria might be biased toward numbers that are too low because the risk for exposure is probably disproportionately distributed among vaccinated and nonvaccinated persons. Another factor contributing to this phenomenon could be the switch in diagnostic techniques, from hemagglutination inhibition and complement fixation tests to ELISA.

Among all European countries, vaccination coverage is highest in Austria, where ≈85% of the total population have received >1 doses of the vaccine (*24*). This high vaccination coverage has led to a dramatic decline in the overall incidence of TBE in Austria, in stark contrast to neighboring countries to the north (Czech Republic) (*11*) and south (Slovenia) (*25*), which have relatively low vaccination rates (>1 doses; data from 2009) of ≈16% and ≈12%, respectively (*14*,*15*). Similar to what was found in a previous study (*24*), the effectiveness of the vaccine for preventing disease is high: 96%–99% after regular vaccination and best-case assumptions. Even among persons with a history of irregular vaccinations, the average protection rate is still >90%. Moreover, vaccine effectiveness is excellent among elderly persons, for whom risk for severe forms of TBE and neuropathologic sequelae is highest (*13*). This high protection rate might be associated with the recent finding that the functional quality of antibodies induced by vaccination is independent of age, although the quantity of antibodies is substantially lower in elderly persons (*34*). Given the reported data on the immunogenicity of TBE vaccines (*22*,*23*) in children, the reason for the slightly lower field effectiveness among children is unclear.

In all 3 countries analyzed in this study, incidence rates for TBE were highest among elderly persons, consistent with increased disease severity and concomitantly lower rates of subclinical infections with increasing age (*5*,*13*). In addition, during the past 10 years, incidence rates have tended to shift toward older age groups. This trend has been especially striking in the Czech Republic (Figure 3), where elderly persons, compared with those in other countries, were underrepresented from 1990 through 1999 (and for whom incidence rates were lower than for adolescents and young adults); however, in the past decade, incidence of TBE among elderly persons has sharply increased. This development probably reflects the ever-improving situation—including health status—of retired persons, which results in increased physical outdoor activities and, thus, risk for tick exposure (*11*). In the Czech Republic and Slovenia, TBE incidence among children and adolescents occurred in distinct peaks, similar to what was observed in Austria during the prevaccination era (*26*). This peak has completely disappeared in Austria (Figure 3), indicating a high degree of protection, especially among groups of young persons, who have the highest risk for exposure. School vaccination programs probably contributed to this achievement. Nevertheless, because vaccination coverage rates (A. Essl, unpub. data) and protection rates (Table) are similar among persons in different age groups in Austria, one would have expected a higher TBE incidence rate among nonvaccinated children in this country during the past 20 years. The factor or factors responsible for the phenomenon observed are currently unknown and might be associated with differences in age-specific risk for exposure in each of the 3 countries. In summary, the example from Austria indicates that TBE vaccination is an excellent way to prevent disease in all age groups. In this country during 2000–2011, vaccination prevented >4,000 cases of TBE.
